# Pediatric Emergency Medicine Simulation Curriculum: Bacterial Tracheitis

**DOI:** 10.15766/mep_2374-8265.10946

**Published:** 2020-08-26

**Authors:** Vaidehi Pidaparti, Ashley Keilman, Jennifer Case, Anita Thomas

**Affiliations:** 1 Resident, Department of Pediatrics, University of Washington School of Medicine and Seattle Children's Hospital; 2 Assistant Professor, Department of Pediatric Emergency Medicine, University of Washington School of Medicine and Seattle Children's Hospital

**Keywords:** Tracheitis, Pediatrics, Emergency Medicine, Stridor, Nurse/Nurse Practitioner, Physician, Physician Assistant, Respiratory Therapist, Pediatric Emergency Medicine, Simulation

## Abstract

**Introduction:**

Pediatric bacterial tracheitis is a rare but life-threatening upper airway infection with mortality rates estimated as high as 20%, typically affecting children between 6 months and 12 years old. Given such high mortality rates, we felt it was important to train medical personnel to evaluate and manage this condition.

**Methods:**

This simulation-based curriculum was developed for health care professionals involving the evaluation and management of an 8-year-old male with symptoms of fever, stridor, worsening barking cough, and increased work of breathing. Critical actions included identifying stridor and airway respiratory distress; monitoring and supporting airway, breathing, and circulation; administering racemic epinephrine and dexamethasone; and identifying and treating bacterial tracheitis as the underlying cause. Scenario-specific debriefing tools were put together to elicit scenario feedback and aid in formative learning.

**Results:**

The scenario was conducted with six fellows and 12 residents and medical students. Per the survey data, the case was rated as highly relevant (median = 5) and highly realistic (median = 5) by participants on a 5-point Likert scale.

**Discussion:**

Pediatric bacterial tracheitis is a low frequency, but high-risk scenario that was amenable to simulation as an educational modality and was well-received by participants. The debriefing tools were implemented as a means of helping instructors customize the scenario for learners based on respective educational backgrounds and learning styles.

## Educational Objectives

By the end of this activity, learners will be able to:

1. Assess and emergently manage airway, breathing, and circulation in a pediatric patient with stridor.
2. Recognize the need for racemic epinephrine and dexamethasone in order to maintain the airway.
3. Formulate a list of possible diagnoses and effectively prioritize elements of evaluation.
4. Identify bacterial tracheitis as a likely cause of symptoms.
5. Demonstrate effective team leadership, frequent reassessment after interventions, team dynamics, and communication.

## Introduction

Bacterial tracheitis is a rare but rapidly life-threatening upper airway infection with mortality rates reported as high as 20%.^1^ It typically affects children between 6 months and 12 years old and the most common causative agent is staphylococcus aureus.^2^ Other less common agents include haemophilus influenzae, streptococcus pyogenes, streptococcus pneumoniae, moraxella catarrhalis, and branhamella catarrhalis.^2^ Infants and children with tracheitis present most acutely with signs and symptoms of airway obstruction or impending respiratory failure, or a combination of the two.^3^ Presenting symptoms may include stridor, hoarse voice, tachypnea, increased nasal or oral secretions, fever, or cough. Patients may also be overall well-appearing or more somnolent if they are hypercarbic or hypoxemic. Infants and children often may have signs and symptoms of other concomitant infections, such as pharyngitis, pneumonia, sinusitis, or otitis media. Some patients may also have a history of gastroesophageal reflux and aspiration, which may predispose them to recurrent tracheitis.^3^ A combination of history and physical exam findings can lead to the diagnosis of bacterial tracheitis, with increased suspicion when a patient does not improve with racemic epinephrine and/or systemic corticosteroids.^1^ Neck radiographs can help to differentiate tracheitis from other causes of upper airway obstruction, showing irregular borders of the tracheal margins and haziness of the tracheal column.^1^ Definitive diagnosis can be made with direct laryngoscopy, showing tracheal erythema and edema, mucopurulent tracheal secretions, and pseudomembranes.^3^ Gram stains, aerobic and anaerobic bacterial cultures, and sensitivities of tracheal secretions are performed to help direct antimicrobial therapy.^1^

As tracheitis can cause acute respiratory decompensation, it requires rapid identification and intervention to properly manage the airway. It is integral, therefore, for providers to form a quick clinical assessment based on history and exam findings and integrate this with pertinent diagnostic test results (neck X-rays if obtained) in developing a disposition plan and preparing for resuscitation. The standard interventions for stridor include racemic epinephrine and systemic corticosteroids, and as aforementioned are not effective in treating tracheitis. Bacterial tracheitis can be treated with broad-spectrum IV antibiotics, but it is imperative that a plan to manage and frequently assess airway, breathing, and circulation occurs concurrently.

This scenario was designed for learners with a foundation in resuscitation knowledge and pediatric advanced life support (PALS)^4^ algorithms, including pediatric emergency medicine fellows, residents, and medical students. Given that pediatric bacterial tracheitis is rare but requires immediate critical interventions that deviate from standard croup management, it is important that learners are exposed to this infection via simulation as a means of expanding their clinical diagnostic and management skills. Thus, because pediatric bacterial tracheitis is a high-risk, low-frequency scenario, it is amenable to simulation as a curriculum. Likewise, participation in this scenario may help providers to increase their comfort with resuscitation practices and broaden their differential with respect to causes of stridor in pediatric patients.

There are currently simulation cases available in *MedEdPORTAL* about causes of acute stridor and upper-airway obstruction, but none specifically addressed bacterial tracheitis as the focus of a simulation case. Bacterial tracheitis management was not discussed in detail in prior stridor cases, thus we felt this curriculum provided a medium to the practice management of this rare but life-threatening pediatric emergency that requires time-sensitive critical interventions. This case is flexible in that it may be used independently or in a series with simulation cases involving acute airway obstruction or stridor.^5–7^ This curriculum may also be used in series with other simulation-based curricula from the pediatric emergency medicine simulation curriculum^8–24^ as it involved core pediatric emergency medicine skills, but may also be used independently, depending on learners’ needs. This curriculum was originally developed for a target audience of pediatric emergency medicine fellows and pediatrics residents, but would also be appropriate for family medicine and emergency medicine residents as well as interdisciplinary simulations involving pediatric emergency physicians, fellows, residents, medical students, nurses, advanced practice providers, pharmacists, and respiratory therapists. It was designed utilizing a similar conceptual framework to the pediatric emergency medicine simulation curriculum cases^8–24^ as mentioned above. This curriculum allowed for evaluation by reaction and learning via Kirkpatrick's model of learning.^25^ The evaluation form and debriefing immediately after the scenario allowed for facilitators to formatively assess what the team of learners learned through the scenario, as well as tailor the scenario for future iterations.

## Methods

### Development

This simulation case was designed by pediatric emergency faculty who have experience with curriculum development and simulation learning, and was based on a real patient case. Learners who participated in the simulation were tasked with rapidly assessing the patient, interpreting physical exam findings, and providing necessary interventions for bacterial tracheitis. Prerequisite knowledge included competency in PALS algorithms.

### Equipment/Environment

The case was based in an emergency room (ED); it can also be conducted in a simulation lab functioning as an ED room or another in situ room. During implementation of the scenario, a high-fidelity child mannequin was used. Participants had access to equipment and medications that are usually found in the ED setting, as well as to medications necessary for this case in particular. The patient was initially sitting on a hospital stretcher, interactive and alert, while connected to monitors. The bedside nurse initiated the scenario by asking the providers to evaluate the patient. A parent provided additional history if asked; depending on participant numbers, the facilitator or embedded participant can stand in as the parent. The team was given a history that the patient was an 8-year-old male presenting with stridor, 1-day history of barking cough that was worsening, and difficulty breathing. Neck X-ray findings were made available to participants on request, as well as chest X-ray findings. As the learners progressed through the scenario, the facilitator provided physical exam findings and clinical updates.

If using a low-fidelity mannequin that cannot demonstrate all physical exam findings, it is appropriate to verbally provide vital signs and physical exam findings (e.g., verbally imitating croup).

### Personnel

The simulation case was able to accommodate four to eight trainees including fellows, residents, or medical students. The simulation is also designed such that it can be run in an interdisciplinary setting with attending and resident physicians, nurses, and respiratory therapists. If participants from multiple disciplines are present, it is ideal that they fill the roles that are closest to their actual clinical roles. Facilitators were comfortable operating the child-sized mannequin and the simulation software. In addition, they were well-versed in facilitation and debriefing methodology. Participants were provided with an orientation to the simulator prior to the case if they had not previously worked with the mannequin.

### Implementation

The simulation sessions occurred during standard pediatric emergency medicine fellow education in 1-hour blocks or during 30-minute resident and medical student teaching blocks. Approximately 5 minutes were spent orienting trainees to the activity, 10–20 minutes participating in the scenario, and 15–35 minutes debriefing depending on the available time. Simulations were either conducted inside patient care rooms within the ED or inpatient floor, or within a simulation lab. A separate space was used to debrief when available. The scenario began by telling participants that an 8-year-old boy was being roomed with stridor, barking cough, and difficulty breathing. His parent described a history of clear nasal rhinorrhea and progressively worsening barking cough that did not improve with steam bath. They also revealed that he has had croup many times in the past. Exam was notable for tachycardia, intermittent dyspnea, raspy voice, stridor, and subcostal retractions. Participants were expected to evaluate the patient, administer racemic epinephrine and steroids, order neck X-ray given changing exam, and think through a comprehensive differential diagnosis. The case concluded when they identified X-ray findings concerning for bacterial tracheitis and administered the appropriate medical intervention of IV antibiotics.

Facilitation of the scenario and debriefing was led by pediatric emergency medicine physicians with training in simulation. Simulation technicians were used (Appendix A) to plan and run the scenario using a high-fidelity pediatric mannequin. Appendix B includes a complete list of recommended equipment and medications commonly found in the ED setting. Facilitators were provided with a critical action checklist (Appendix C) to review during the case to ensure key objectives were met. Diagnostic test results used in the case are included in Appendices A, D, and E. They were also given a glossary of communication tools in Appendix F (as adapted from a prior pediatric emergency medicine simulation curriculum *MedEdPORTAL* publication^8^) to review prior to the session as a means of establishing a cohesive framework around teamwork. This glossary can also be provided to learners as a resource prior to or after the scenario. We used the materials contained in Appendix G to facilitate discussion and provide constructive feedback during the debrief portion. Key concepts in diagnosis and management were reviewed with participants after the scenario to help solidify what they had learned so that they were better equipped to recognize the presentation of and appropriately manage bacterial tracheitis. Didactics were provided in the form of a teaching handout (Appendix H) during the debriefing, but can be provided prior to the simulation depending on the learners’ needs. Appendix H was created as a teaching handout/quick reference for learners from three references.^1,3,26^ We used an evaluation form (Appendix I) to elicit feedback from participants and fine tune the simulation for further iterations.

### Debriefing

We used the tools in Appendix G to assist in facilitating debrief sessions after the simulations. This tool provided facilitators with a means of tailoring discussions based on the needs and performance of the participants. We began the debrief by allowing participants to provide both general impressions and respective experiences followed by a joint discussion of the components of the scenario. Reflections and observations from participants were then used to help lead into conversations about teamwork and communication as well as reinforcing diagnostic and management skills.

### Assessment

The scenario was facilitated by experienced pediatric emergency medicine faculty who provided content expertise and constructive feedback to learners on their respective performances as relating to the learning objectives. All participants completed a survey after the completion of the debrief sessions. Participants were asked to state their agreement with evaluative statements using a 5-point Likert scale (1 = *strongly disagree*, 2 = *disagree*, 3 = *neutral*, 4 = *agree*, 5 = *strongly agree*). They were asked about their experiences during the educational simulation and about their respective clinical comfort related to the learning objectives after participating in the session. They were also asked to answer free response questions regarding their experiences.

## Results

We implemented this curriculum with six pediatric emergency medicine fellows, ranging from first- to third-year fellows, and 12 PGY 1–3 residents (pediatrics and family medicine) and third- and fourth-year medical students, for a total of 18 participants. We used Kirkpatrick's model level 1, or reaction, as evaluations were filled out immediately after the debriefing.^25^ Participants’ agreement with statements related to their experiences during the simulation sessions are summarized in Table 1 using a 5-point Likert scale (1 = *strongly disagree*, 2 = *disagree*, 3 = *neutral*, 4 = *agree*, 5 = *strongly agree*). Table 2 summarized participants’ confidence with skills and knowledge after taking part in the sessions using the same 5-point Likert scale.

**Table 1.**
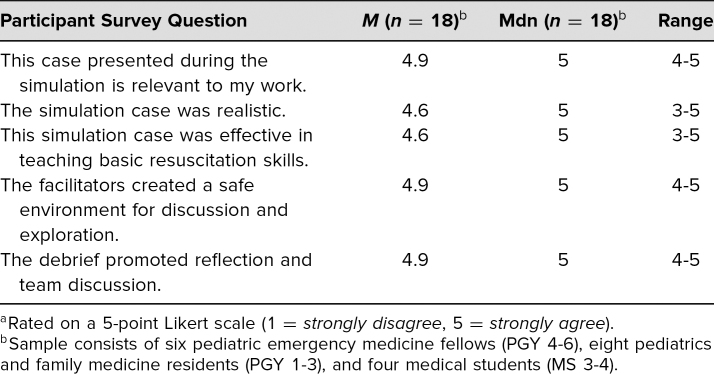
Participant Experience During the Simulation Session^a^

**Table 2.**
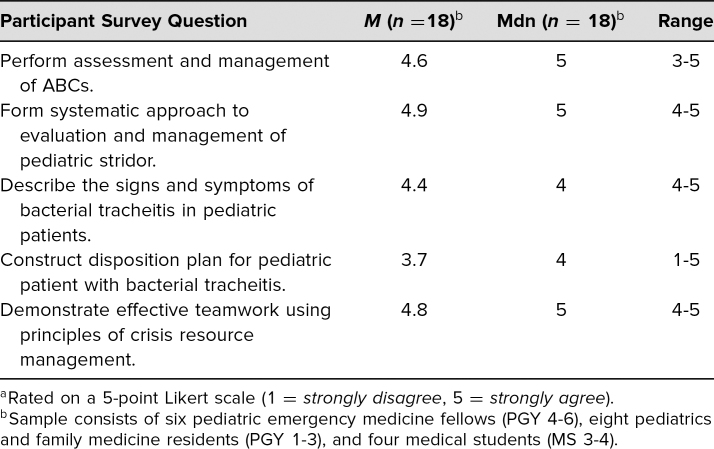
Participant Clinical Confidence After Session^a^

Additionally, during the debriefing, participants were able to verbally provide feedback that not only informed subsequent iterations of the simulation curriculum, but also allowed facilitators to gauge the team's knowledge of bacterial tracheitis. Kirkpatrick's level 2, or learning, can be elicited when facilitators are debriefing and specifically discussing the case learning objectives.^25^ Additionally, many formally trained simulation instructors conclude debriefings by asking learners to state one piece of learning from the case. This simulation, however, was designed to be formative rather than summative for the team of learners.

Participants were asked to list ways in which the simulation session would modify their clinical decision-making and how the scenario could be improved upon after partaking in the scenario. Open-ended feedback is summarized below:

• “Exposure to case increased my depth of understanding of how to approach and consider a broader differential in pediatric patients with stridor.”
• “I feel more comfortable performing more effective airway management and would consider such strategies as obtaining lateral neck films early or intubation in the operating room in the future.”
• “The scenario encouraged me to consider bacterial tracheitis in pediatric patients with stridor and re-evaluate if first like treatments (i.e., racemic epinephrine) were not improving symptoms.”
• “The scenario reinforced the importance of communication and teamwork, especially when dealing with potential emergencies.”
• “It would have been more helpful to further discuss the differential diagnosis in pediatric patients with stridor and explore what would happen once the patient left the ED.”

## Discussion

The purpose of this simulation case was to provide an environment for learners to practice management of patients using PALS algorithms while encouraging them to broaden their differential diagnosis when thinking about stridor in pediatric patients. It was also intended for learners to recognize and manage respiratory distress, as well as to consider imaging modalities such as X-ray to assist with diagnosis. Bacterial tracheitis should be considered for a pediatric patient with stridor who is refractory to usual management, as it requires different management than the common therapies for pediatric stridor. This scenario facilitated learning by means of allowing providers to work as a team and use their clinical judgment to try different management strategies in a controlled learning environment. Conducting simulation sessions with five to eight learners per session would allow each learner to assume an active role in the scenario. If resuscitation teams are typically larger or smaller in size, the number of participants per session may be adjusted to reflect local practice. If there are additional participants they could be assigned to specific observer roles and invited to comment on aspects of the case during the debrief in order to maximize their engagement with the session. Team leadership, dynamics, and communication may be assessed by the facilitator using the teamwork and communication evaluation guide in Appendix G and discussed during the debrief to ensure that participants recognized areas of successful teamwork and opportunities for further improvement during future resuscitations.

### Limitations

Limitations in the evaluation of this educational activity included that the scenario was mainly tested on pediatric emergency medicine fellows, pediatrics residents, family medicine residents, and third- and fourth-year medical students. However, this scenario is applicable to all health care personnel who may take care of an acutely ill pediatric patient with tracheitis, with all of whom the scenario was not specifically trialed. Thus, there may be aspects of this case which could be adapted to better serve other populations of health care workers, including physicians of other specialties, nursing students, advanced practice providers and nurses who also care for this patient population. Mannequin limitations included not being able to simulate tracheal tug on the mannequin or simulate deep retractions, thus limiting the realism of the scenario. This may be mitigated by providing participants with a video clip of a pediatric patient in severe respiratory distress. This curriculum was assessed primarily via Kirkpatrick's level 1, as mentioned in the results and introduction method, but does not delve into Kirkpatrick's level 2 (learning) to the same degree.^25^ To address this, performance could be measured as summative learning or Kirkpatrick level 2 if this simulation was repeated with the same group of learners multiple times over a prolonged time period, but it was not feasible to perform pre- and posttesting on all the same learners to assess performance/knowledge. Additionally, we used a convenience sample of providers at our participating institutions, which may limit generalizability. However, given that the scenario was trialed with varying levels of adult learners (third-year medical students through to pediatric emergency medicine fellows), the results are likely generalizable to the learner populations at other institutions. Translation of knowledge acquired from this session to actual clinical resuscitations was not measured by our evaluation tool as this was a high-risk but infrequently encountered patient presentation.

### Future Implications

In terms of areas for improvement, it was frequently expressed by residents and medical students that they did not feel as confident about developing a robust disposition plan and wanted to have more teaching regarding the differential diagnosis for causes of stridor. We attempted to be more transparent in a subsequent iteration and we have developed interventions that can be used to scaffold trainees’ learning and allow them to build a stronger knowledge base. One strategy would be to provide a published review article as a prereading so that learners feel more prepared coming into the simulation case.^1^ Likewise, an embedded participant can be provided with a list of scripted suggestions to give participants if they are having trouble proceeding through the scenario. The debrief portion is also amenable to change; specifically, facilitators can dedicate more time reviewing the differential diagnosis for stridor and the management of bacterial tracheitis, as well as potential outcomes of bacterial tracheitis depending on whether treatment is obtained or not.

## Appendices

Bacterial Tracheitis Simulation Case.docxEnvironmental Preparation.docxCritical Action Checklist.docxSoft Tissue Neck X-Rays.docxChest X-ray.docxCommunication Glossary.docxDebriefing Guide.docxTeaching Handout.pdfEvaluation Form.docx
All appendices are peer reviewed as integral parts of the Original Publication.
