# Supplementary material for: Pediatric Emergency Medicine Simulation Curriculum: Bacterial Tracheitis
Source: MedEdPORTAL. 2020 Aug 26;16:10946. doi: 10.15766/mep_2374-8265.10946 (PMC7449579; doi:10.15766/mep_2374-8265.10946)
Supplement: Supplementary file 1 — Bacterial Tracheitis Simulation Case.docxEnvironmental Preparation.docxCritical Action Checklist.docxSoft Tissue Neck X-Rays.docxChest X-ray.docxCommunication Glossary.docxDebriefing Guide.docxTeaching Handout.pdfEvaluation Form.docx [file mep_2374-8265.10946-s001.zip › A. Bacterial Tracheitis Simulation Case.docx]

| **Appendix A: Tracheitis Simulation Case**  **SIMULATION CASE TITLE: Tracheitis**  **AUTHORS: Vaidehi Pidaparti, MD, Ashley Keilman, MD, Jennifer Case, MD, Anita Thomas, MD, MPH** | |
| --- | --- |
| **PATIENT NAME: Evan**  **PATIENT AGE: 8 year old**  **CHIEF COMPLAINT: Worsening barking cough, retractions, inspiratory stridor** | |
|  | |
| **Brief narrative description of case**  *Include the presenting patient chief complaint and overall learner goals for this case* | An 8 yo boy is brought in with worsening barky cough, retractions, and inspiratory stridor in the setting of 1 day history of mild rhinorrhea. Mother initially attributed symptoms to croup and placed him in steam bath with minimal improvement in symptoms. The child was then brought to the emergency department. No interventions were administered prior to arrival to the emergency department.  Anticipated interventions include initial assessment, identification and management of stridor, potentially difficult airway, and bacterial tracheitis. Anticipated interventions include supporting airway, breathing, and circulation (ABCs), identifying stridor and administering racemic epinephrine and dexamethasone, and identifying possibility of bacterial tracheitis and starting IV antibiotics. The team should anticipate potential for patient to decompensate and the need to manage the airway and perform intubation or transfer to the OR for further airway management. |
| **Primary Learning Objectives**  *What should the learners gain in terms of knowledge and skill from this case? Use action verbs and utilize Bloom’s Taxonomy as a conceptual guide* | 1. Demonstrate ability to assess and emergently manage airway, breathing, and circulation in a pediatric patient with stridor. 2. Formulate a list of possible diagnoses and prioritize elements of evaluation 3. Identify need for racemic epinephrine and dexamethasone in order to maintain airway. 4. Identify bacterial tracheitis as likely cause of symptoms. 5. Develop and execute a management plan for a patient with bacterial tracheitis. 6. Demonstrate effective team leadership, reassessment after interventions, team dynamics and communication |
| **Critical Actions**  *List which steps the participants should take to successfully manage the simulated patient. These should be listed as concrete actions that are distinct from the overall learning objectives of the case.* | Clinical State #1: Presentation   1. Complete primary and secondary assessment    1. Place patient on monitors    2. Assess need for airway intervention 2. Collect focused history. 3. Identify stridor and administer racemic epinephrine and dexamethasone. 4. Develop differential diagnosis for stridor. 5. Repeat assessment after first dose of racemic epinephrine and dexamethasone. 6. Obtain IV/IO access   Clinical State #2: Clinical Progression/Stabilization   1. Recognize worsening respiratory state after first dose of racemic epinephrine and steroids. 2. Recognize need for continued racemic epinephrine. 3. Recognize need for third dose of racemic epinephrine. 4. Repeat assessment and broaden differential. 5. Consider alternative diagnoses such as bacterial tracheitis, peritonsillar abscess, retropharyngeal abscess, epiglottitis, airway foreign body. 6. Order neck x-ray and identify radiologic signs of tracheitis. 7. Identify need for potential support from otolaryngology, Anesthesiology, PICU. 8. Start antibiotics for bacterial tracheitis. 9. Determine disposition - floor vs. ICU |
| **Learner Preparation**  *What information should the learners be given prior to initiation of the case?* | A previously healthy, fully immunized 8 year old male with history of multiple episodes of croup is brought in to the emergency department by his mother for evaluation of worsening barking cough, difficulty breathing. The symptoms began in the middle of the night on the day of presentation. Mother placed him in a steam bath just before bringing him to the emergency department with minimal improvement in symptoms. He developed clear rhinorrhea, barking cough, and intermittent difficulty breathing one day ago. Patient remained awake and alert during drive to ED. No interventions were given. |

| **Initial Presentation** | | | |
| --- | --- | --- | --- |
| **Initial vital signs** | HR 140 bpm, BP 112/70, RR 32, T 38C, O2 Sat 95%, Wt 25 kg | | |
| **Overall Appearance**  *What do learners see when they first enter the room?* | Male patient awake, alert, somewhat pale, sitting on bed, intermittently dyspneic with raspy voice, talking to mother | | |
| **Actors and roles in the room at case start**  *Who is present at the beginning and what is their role? Who may play them?* | Triage nurse brings back patient to room immediately with concern for croup, calls team into room to assess patient. Learners may assign themselves to role. The facilitator can play the role of the parent. | | |
| **HPI**  *Please specify what info here and below must be asked vs what is volunteered by patient or other participants* | Evan is an 8 year old male. He first started to have barking cough, clear nasal secretions the day prior to presentation. This evening, his mom noticed that he was having worsening barking cough and difficulty breathing. She placed him in a steam bath but this did not help with his symptoms. She then brought him to the emergency department. He has had croup many times and been treated with steroids. He is otherwise healthy.  If asked: Difficulty breathing refers to subcostal retractions. Mom has also noted that his voice has changed since yesterday. No fever, nausea, vomiting, diarrhea, rashes. | | |
| **Past Medical/Surgical History** | **Medications** | **Allergies** | **Family History** |
| Multiple past episodes of croup  Immunizations up to date (all must be asked) | None  (must be asked) | Tree nuts (must be asked) | None (must be asked) |
| **Physical Examination** | | | |
| **General** | Awake, alert, talking, somewhat pale appearing | | |
| **HEENT** | Normocephalic, atraumatic, PERRLA, 3+ tonsils without exudate with symmetric tonsillar pillars, Uvula is midline | | |
| **Neck** | Full range of motion | | |
| **Lungs** | Subcostal retractions, Intermittently dyspneic with raspy voice, Able to count to 5 before taking a breath, Rare end expiratory wheezing at bases | | |
| **Cardiovascular** | Regular, tachycardic, no murmur, 2+ pulses, cap refill 2-3 seconds | | |
| **Abdomen** | Soft, non-tender, non-distended, no organomegaly, normal bowel sounds | | |
| **Neurological** | Awake, alert, moves all extremities, no focal deficits, GCS 14 (following most but not all commands) | | |
| **Skin** | Warm, dry, no rash | | |
| **GU** | Exam deferred | | |
| **Psychiatric** | Cooperative | | |

| **Instructor Notes - Changes and CASE Branch Points** | | |
| --- | --- | --- |
| **Intervention / Time point** | **Change in Case** | **Additional Information** |
| Learners establish team roles (done prior to or at the beginning of scenario) |  | Roles:  Team lead MD  Airway MD  Survey MD  Bedside RN |
| Patient is on gurney with parent at bedside | Vitals: HR 140 bpm, BP 112/70, RR 32, T 38.0 C, O2 Sat 95%, Wt 25 kg | Bedside RN placing monitors on pt |
| 5 minutes into the case | Decision to administer racemic epinephrine and dexamethasone | If no racemic epinephrine or steroids are given, facilitator or embedded participant acting as parent states “he has gotten steroids and mask treatments for croup in the past” |
| 10 minutes into the case | After first dose of racemic epinephrine and steroids, stridor does not improve and he develops diffuse retractions. He looks more panicked and becomes more tachypneic and tachycardic with PIV placement. |  |
| Repeat vitals after first dose of racemic epinephrine | RR 38, HR 150, BP 120/80, SpO2 93% on room air, T 39.0 C | Some advanced learners (such as pediatric emergency medicine fellows) may begin setting up for endotracheal intubation. If learners attempt to proceed with intubation, the facilitator or embedded participant should state “should we try additional doses of racemic epinephrine before proceeding to intubation?” Facilitators may choose to have an embedded participant also verbalize “Should we think about contacting otolaryngology or the OR or the PICU prior to intubating?” Alternatively, facilitators may opt to allow learners to attempt intubation or use adjunct airways, bearing in mind that the team should utilize smaller diameter ETT (would expect to intubate a 25 kg patient with a 6.0 cuffed ETT, but would set up a 5.5, 5.0, and 4.5 cuffed ETTs, but may vary based on your mannequin) |
| Second dose of racemic epinephrine is given | Mild improvement in dyspnea and severity of retractions  RR 35, HR 148, BP 120/80 SpO2 94% on room air, T 39.0 C |  |
| Team requests peripheral intravenous (PIV) line placement | Slight worsening of dyspnea and retractions with PIV placement/agitation  RR 40, HR 155, BP 135/80 SpO2 96% on room air, T 39.0 C | Facilitator may have an embedded participant question PIV placement and state “Won’t placing a PIV upset the patient and worsen the stridor and potentially occlude the airway?” To which a team member should acknowledge the embedded participant’s concern, but state that the patient requires a PIV for adequate treatment, that the team will monitor him and feel that he can handle it without significant respiratory distress. |
| If third dose of racemic epinephrine is given | Stridor improves slightly but is still present, retractions improve but are still present though the patient is able to speak in full sentences. Intermittent barky cough. Rare intermittent expiratory wheezes at bases. |  |
| Repeat vitals after third dose of racemic epinephrine | Tachypnea improves to 24; sinus tachycardia to 135 on monitor, BP 118/78, SpO2 98% on room air, T 38.0 C |  |
| Participant requests neck X-ray and or Chest x ray | Neck x-ray read (if asked for): Slightly asymmetric subglottic narrowing with faint irregular soft tissue filling defects on lateral view; concerning for exudative/membranous tracheitis.  Chest x-ray (if asked for): No focal opacities, viral perihilar markings, normal cardiac silhouette. | May provide Appendix D: Bacterial Tracheitis Simulation Neck Soft Tissue X-rays  May provide Appendix E: Bacterial Tracheitis Simulation CXR |
| Team requests antibiotics/intravenous fluid administration/anti-pyretic | Vital signs: RR 20; HR: 135,, BP 118/78, SpO2 98% on room air, T 38.0 C |  |
| If an otolaryngology/anesthesia/pediatric intensive care (PICU) consult is requested | Consultant is en route to the Emergency Department and will arrive in 5-10 minutes |  |

**Ideal Scenario Flow**

A triage nurse (embedded participant or facilitator) will ask participants to come into the room to examine the patient. The triage nurse may also call in one participant who then recruits additional help after assessing the patients. Participants connect bedside monitors to the patient and perform an initial assessment. They recognize stridor and subcostal retractions, intermittent dyspnea, raspy voice and ability to count to 5 before taking a breath. They promptly order racemic epinephrine and dexamethasone to treat presumed croup. If the participants stop after 1 dose of racemic epinephrine, his stridor will worsen and he will look more panicked, have worsening diffuse retractions, tachypnea, tachycardia, and develop fever to 38.0C. The patient will show a modest response to a second dose of racemic epinephrine but have persistent respiratory distress and stridor at rest. If the participants give a third dose of racemic epinephrine, the patient will respond with resolution of stridor, improving tachypnea, improving retractions, and ability to speak in full sentences. He will continue to have intermittent barking cough and rare intermittent expiratory wheezes at bases. After administering the third dose of racemic epinephrine the team should promptly reassess the patient’s airway and recognize the need to broaden the differential diagnosis to bacterial tracheitis, peritonsillar abscess, retropharyngeal abscess. The team should recognize the need for potential ENT, anesthesia, or PICU consultation and should be prepared for the patient’s airway to become difficult and require either intubation or OR management.

**Anticipated Management Mistakes**

1. Premature diagnostic closure: Some participants may diagnose the patient too early and assume that he has croup due to presenting symptoms, history of multiple episodes of croup that were responsive to steroids, overall non-toxic appearance. As such, a broader differential may not be properly considered, neck x-ray may not be obtained, and IV antibiotics may not be started.
2. Failure to properly reassess airway after intervention: Participants may not promptly reassess patient after each medication is administered to determine need for additional interventions. If team proceeds to invasive support (i.e. intubation) prior to maximizing medical therapy they may be prompted by the facilitator or embedded participant. If intubation is deemed necessary, participants should recognize need to consider subspecialty consultation including otolaryngology and anesthesia due to difficult airway.

1. Failure to administer antibiotics: Participants may not administer antibiotics to treat for bacterial tracheitis. If this occurs, they should be prompted by the facilitator or embedded participant to consider antibiotic administration.
