# Supplementary material for: Pediatric Emergency Medicine Simulation Curriculum: Bacterial Tracheitis
Source: MedEdPORTAL. 2020 Aug 26;16:10946. doi: 10.15766/mep_2374-8265.10946 (PMC7449579; doi:10.15766/mep_2374-8265.10946)
Supplement: Supplementary file 1 — Bacterial Tracheitis Simulation Case.docxEnvironmental Preparation.docxCritical Action Checklist.docxSoft Tissue Neck X-Rays.docxChest X-ray.docxCommunication Glossary.docxDebriefing Guide.docxTeaching Handout.pdfEvaluation Form.docx [file mep_2374-8265.10946-s001.zip › B. Environmental Preparation.docx]

**Appendix B:** Bacterial Tracheitis Simulation Environment Preparation

Before each simulation, ensure the anticipated resuscitation equipment is available for the team’s use. The medications and equipment available should reflect what is actually available to participants in real practice. Not all medications or equipment are necessary for the simulation, as anticipated with ideal flow, but reflect what is often found in a pediatric emergency medicine department for the care of critically ill patients.

**Resources**

PALS reference cards, material

Patient Weight Estimator such as a Broselow tape

Pediatric Resuscitation Medication references (e.g.: Broselow tape, reference cards)

Documentation forms

**Personal Protective Equipment Universal Precautions**

Staff gowns

Gloves

Mask and face shields

**Simulated Medications (consider having all or only a limited number of medications available; those bolded are expected to be used in this simulation)**

**Acetaminophen**

Adenosine

Amiodarone

**Ampicillin-sulbactam**

Atropine

**Cefotaxime**

Ceftazidime

**Ceftriaxone**

**Clindamycin**

Epinephrine 1:10,000

**Epinephrine 1:1,000 for racemic use**

Etomidate

Fentanyl

Ketamine

Lidocaine

Lorazepam

Midazolam

Morphine

Norepinephrine

**Normal Saline/Lactated Ringers**

Ibuprofen

Procainamide

Rocuronium

Sodium bicarbonate

Succinylcholine

**Vancomycin**

**Equipment (consider having some or all equipment based on usual clinical environment)**

Simulator manikin in hospital gown or clothing, on bed with patient identification band

Monitor – Noninvasive blood pressure (NIBP), Heartrate (HR), Respiratory Rate (RR), Oxygen saturation (SpO2), temperature(T) and end-tidal carbon dioxide (ETCO2)

Blood Pressure cuff, Heart Rate monitor leads, oxygen saturation probe, defibrillator cables and ETCO2 cannula

Oxygen hook-up - on wall or cylinder

Bag-valve-mask system, multiple size masks

Oxygen (O_2_ )– nasal cannula, mask - simple and/or non-rebreather

Suction device

Thermometer, temperature probe

Nasal, and oral airways, multiple sizes

Shoulder roll

Endotracheal tubes- 3.0, 3.5, 4.0, 4.5, 5.0, 6.0, 6.5, 7.0, 7.5 cuffed, stylets

Laryngoscope, Miller and Mac blades, multiple sizes

End-tidal CO_2_ colorimeter

Nasogastric tube(s)

Stethoscopes

IV/Angiocath, various sizes

IO needles, 2 sizes

Gauze, Tape

IV tubing/blood product tubing and filters

IV pumps, pressure bags/ blood product pumps

Syringes, multiple sizes

Bedside blood sample processors: glucose, electrolytes, gases

Specimen tubes

Code cart

CPR stool, backboard

Defibrillator / AED

High flow nasal cannula system and/or nasal CPAP system
