# Supplementary material for: Pediatric Emergency Medicine Simulation Curriculum: Bacterial Tracheitis
Source: MedEdPORTAL. 2020 Aug 26;16:10946. doi: 10.15766/mep_2374-8265.10946 (PMC7449579; doi:10.15766/mep_2374-8265.10946)
Supplement: Supplementary file 1 — Bacterial Tracheitis Simulation Case.docxEnvironmental Preparation.docxCritical Action Checklist.docxSoft Tissue Neck X-Rays.docxChest X-ray.docxCommunication Glossary.docxDebriefing Guide.docxTeaching Handout.pdfEvaluation Form.docx [file mep_2374-8265.10946-s001.zip › C. Critical Action Checklist.docx]

**Critical Action Checklist**

Clinical State #1: Presentation

1. Complete primary and secondary assessment
   1. Place patient on monitors
   2. Assess need for airway intervention
2. Collect focused history.
3. Identify stridor and administer racemic epinephrine and dexamethasone.
4. Develop differential diagnosis for stridor.
5. Repeat assessment after first dose of racemic epinephrine and dexamethasone.
6. Obtain IV/IO access

Clinical State #2: Clinical Progression/Stabilization

1. Recognize worsening respiratory state after first dose of racemic epinephrine and steroids.
2. Recognize need for continued racemic epinephrine.
3. Recognize need for third dose of racemic epinephrine.
4. Repeat assessment and broaden differential.
5. Consider alternative diagnoses such as bacterial tracheitis, peritonsillar abscess, retropharyngeal abscess, epiglottitis, airway foreign body.
6. Order neck x-ray and identify radiologic signs of tracheitis.
7. Identify need for potential support from otolaryngology, Anesthesiology, PICU.
8. Start antibiotics for bacterial tracheitis.
9. Determine disposition - floor vs. ICU
