# Supplementary material for: Pediatric Emergency Medicine Simulation Curriculum: Bacterial Tracheitis
Source: MedEdPORTAL. 2020 Aug 26;16:10946. doi: 10.15766/mep_2374-8265.10946 (PMC7449579; doi:10.15766/mep_2374-8265.10946)
Supplement: Supplementary file 1 — Bacterial Tracheitis Simulation Case.docxEnvironmental Preparation.docxCritical Action Checklist.docxSoft Tissue Neck X-Rays.docxChest X-ray.docxCommunication Glossary.docxDebriefing Guide.docxTeaching Handout.pdfEvaluation Form.docx [file mep_2374-8265.10946-s001.zip › E. Chest X-Ray.docx]

**Appendix E:** Bacterial Tracheitis Simulation Chest X-Ray (CXR):


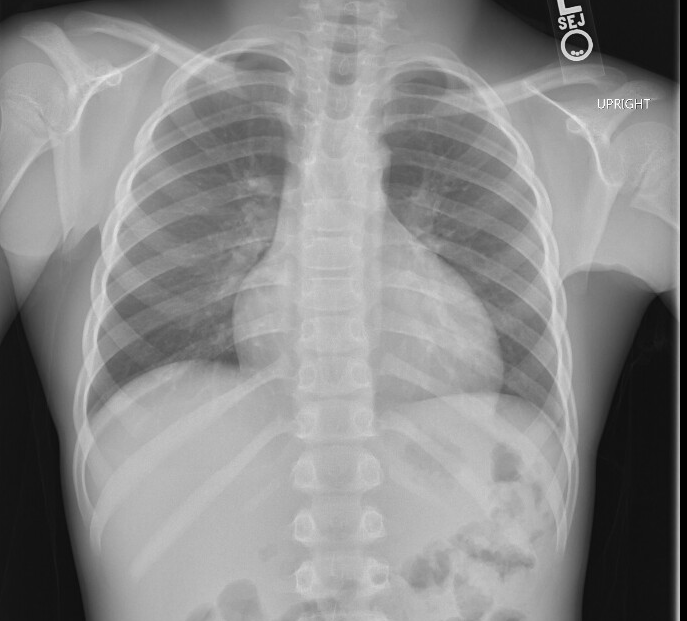


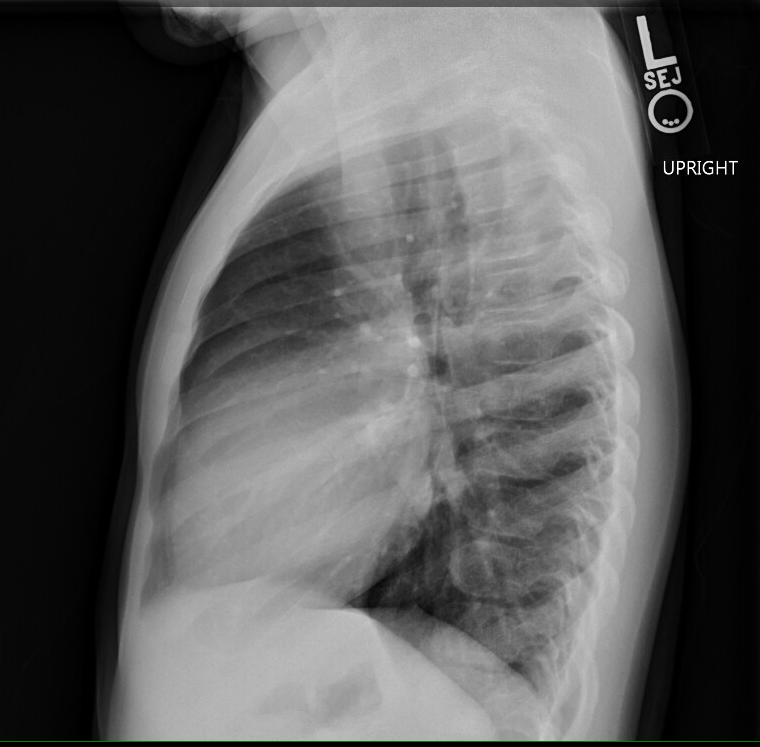


*Images courtesy of Anita Thomas, MD, MPH*
