# Supplementary material for: Pediatric Emergency Medicine Simulation Curriculum: Bacterial Tracheitis
Source: MedEdPORTAL. 2020 Aug 26;16:10946. doi: 10.15766/mep_2374-8265.10946 (PMC7449579; doi:10.15766/mep_2374-8265.10946)
Supplement: Supplementary file 1 — Bacterial Tracheitis Simulation Case.docxEnvironmental Preparation.docxCritical Action Checklist.docxSoft Tissue Neck X-Rays.docxChest X-ray.docxCommunication Glossary.docxDebriefing Guide.docxTeaching Handout.pdfEvaluation Form.docx [file mep_2374-8265.10946-s001.zip › F. Communication Glossary.docx]

**Appendix F**: Bacterial Tracheitis Simulation Communication Glossary

| **Term** | **Definition** |
| --- | --- |
| **Adaptability** | The ability to adjust strategies and alter a course of action in response to changing internal and external conditions. |
| **Call-Out** | A method to communicate critical information during an emergent event. Helps the team prepare for vital next steps in patient care. *(Example: “Airway status?” – “Airway clear”; “Breath sounds?” – “Breath sounds decreased on right”)* |
| **Check-Back or Closed-Loop Communication** | Communication verification of information. The sender initiates the message; the receiver accepts it and restates the message. In return, the sender verifies that the re-statement of the original message is correct or amends if not. (*Example: “Give Dexamethasone 10 mg IV push” – “Dexamethasone 25 mg IV push” – “That’s correct”)* |
| **CUS** | Utilizing the key phrases “I am **C**oncerned, I am **U**ncomfortable, This is a **S**afety Issue” in order to clearly understand the gravity of the issue raised. |
| **Debrief** | Brief, informal information exchange session after an event designed to improve team performance and effectiveness. |
| **Pre-Brief** | Discussion prior to start of simulation/encounter to assign roles, establish expectations, anticipated outcomes, and contingencies. |
| **Shared Mental Model** | An organizing knowledge structure of relevant facts and relationships about a task or situation that is verbalized and held by team members. |
| **Situational Awareness** | The ability to identify, process, and comprehend the critical elements of information about what is happening to the team with regards to the mission. Knowing “What is going around you” and “What is likely to happen next” |

*Adapted from Reid J, Stone K. Pediatric emergency medicine simulation curriculum: hypovolemic shock. MedEdPORTAL. 2013;9:945
