# Supplementary material for: Pediatric Emergency Medicine Simulation Curriculum: Bacterial Tracheitis
Source: MedEdPORTAL. 2020 Aug 26;16:10946. doi: 10.15766/mep_2374-8265.10946 (PMC7449579; doi:10.15766/mep_2374-8265.10946)
Supplement: Supplementary file 1 — Bacterial Tracheitis Simulation Case.docxEnvironmental Preparation.docxCritical Action Checklist.docxSoft Tissue Neck X-Rays.docxChest X-ray.docxCommunication Glossary.docxDebriefing Guide.docxTeaching Handout.pdfEvaluation Form.docx [file mep_2374-8265.10946-s001.zip › G. Debriefing Guide.docx]

**Appendix G**: Bacterial Tracheitis Simulation Debriefing Guide

**Debriefing Overview**

*We believe that reflective learning occurs in the DEBRIEF. It’s an opportunity for learners to reflect on their medical decision making, technical, teamwork and communication skills. The ultimate goal is to identify gaps and potential solutions to close those gaps, leading to improved patient safety and better quality care.*

**Framework for debriefing:**

We model our debriefing after PEARLS.^1^ Each debrief typically has 4 phases:

· **Reactions phase**- opportunity for learners to express their emotional experience, where they may reveal key areas that are important to them

· **Description phase-** opportunity for learners to summarize key events in the scenario to ensure that educators and learners are on the same page

· **Analysis phase-** opportunity to explore the medical decisions, technical, teamwork and communication performance of the team

· **Summary phase-** review of key take home points, led by learners or educator

General Debriefing Goals:

- Create a safe learning environment
  - Share a learning contract (PreBrief)
  - Normalize gaps in performance, if at all possible
- Ask open ended questions (avoid yes/no questions)
- Try to facilitate the team’s discussion (avoid lecturing)

**1) Reactions Phase**

We feel that until emotions are addressed, it’s difficult for adult learners to “move on” to an analysis of their performance and opportunities for improvement. If a group or team member is emotionally charged (e.g. sad, mad or frustrated), it’s usually difficult for the individual or the group to be actively engaged, receptive to feedback and able to promote learning, until the emotions are addressed.

An example: a medication error occurs. One team member may think it is all their fault. They may feel embarrassed, judged, etc. If they can verbalize this, other team members may offer different perspectives, which enables the team to process the error together, potentially identifying contributing systems issues. If the emotions aren’t addressed, separate people may feel embarrassed, responsible and not engage in a discussion, failing to identify systems issues which led to the error.

What you might say:

· “How did that feel?”

· “How did that go?”

· “Initial reactions to the scenario?”

· “How are the rest of you feeling?”

**2) Description phase**

Summary of key events to ensure that educator and participants are on the same page.

What you might say:

· “Could someone summarize the case so we are all on the same page?”

· “From your perspective, what were the main issues you dealt with?

**3) Analysis phase**

Promote reflection on performance (medical decision making, technical skills, teamwork and communication), identify opportunities for improvement.

What you might say:

· “Let’s talk more about the case.”

· “What aspects did your team manage well? Why?”

· “What could your team manage better next time? Why?”

· “I want to spend a couple minutes talking about XXX. Can you tell me more about what was going on?”

· I noticed you [*behavior*]…next time you may want to [*suggested behavior*]… because [*provide rationale*].

### **5) Summary phase**

Opportunity to review key learning points. Participants’ or educator can identify take home points.

What you might say:

*Medical management/technical skills examples:*

· “This was a scenario of a patient with bacterial tracheitis.”

· “Signs and symptoms of bacterial tracheitis include: stridor, respiratory distress, and fever.”

· “Formulating a list of possible diagnoses is critical to identifying an etiology and determining a treatment plan.”

· “Evaluation of stridor includes: evaluating the ABCDE’s and consideration of a neck x-ray.”

· “Management of bacterial tracheitis includes: treatment of ABCDE’s, racemic epinephrine, intravenous antibiotics, and subspecialist involvement with hospitalization as disposition”

*Teamwork/ communication examples:*

- - Recognize need for a full resuscitation team when a patient has a stridor and significant respiratory distress.
  - Designate leadership and team member roles to ensure coordinated team functioning.
  - Role assignment to specific individuals to avoid duplication/omission of tasks
  - Respect toward all team members is key to enable empowerment to speak up if patient safety issues arise.
  - Use brief or huddle to create a shared mental model for the working diagnosis and management plan.
  - Closed-loop communication is of paramount importance to ensure safe and adequate communication.

^1^Cheng A, Grant V, Huffman J, et al. Coaching the debriefer: Peer coaching to improve debriefing quality in simulation programs. Simul Healthc. 2017 Oct; 12(5): 319-25.

**Debriefing Guide**

Below are examples of learning objective based statements & questions you may use to debrief the team.

| **Examples of debriefing for different learning objectives** | | |
| --- | --- | --- |
| **Assess a patient with stridor and respiratory distress** | | |
| Debriefer Script | Reference Material | Instructor Notes |
| I noticed you *(were complete/missed some opportunities)* in performing your initial evaluation- ABCDE’s. This was *(great/could have been even better)* because early identification and management could lead to improved outcomes*.*  · How did your team decide on the evaluation priorities?  · What helped/hindered you?    I saw you *(were quick/ took a while)* to identify bacterial tracheitis in your differential diagnosis. This *(was great/could have been even better)* since delays in recognition can result in clinical deterioration.  · What were you considering in your differential diagnosis?  · What helped/hindered you from considering other options? | Components of an initial evaluation  · Primary assessment (ABCDEs)  · Vital signs  · Secondary assessment    Differential diagnosis for stridor and respiratory distress   - Croup - Bacterial tracheitis - Epiglottitis - Airway foreign body |  |
| **Identify and manage bacterial tracheitis** | | |
| Debriefer Script | Reference Material | Instructor Notes |
| I noticed you *(were quick/took a while)* to identify stridor and administer racemic epinephrine. This was (*great/could lead to delays)* since delays in recognition can result in clinical deterioration.  · What were your thoughts/ priorities?  · What helped/hindered you from identifying bacterial tracheitis?  · How did you determine which medications to give? How did you assess the patient’s response to the medications given? | Initial management of stridor and respiratory distress   - Racemic epinephrine - Steroids - Supplemental oxygen - Consider IV access |  |
| **Reassess breathing after intervention** | | |
| Debriefer Script | Reference Material | Instructor Notes |
| I noticed you *(were quick / could have been quicker)* to obtain a repeat respiratory assessment and repeat set of vital signs when the rhythm changed. This *(was great/could have been better)* because reassessing the patient is key in determining next steps in management.  · What were your thoughts/priorities after you stabilized the patient?  · What helped/hindered you? | Evaluation of patient after racemic epinephrine   - Recognize lack of response to treatment - Broaden differential to include bacterial tracheitis, epiglottitis, airway foreign body |  |

| **Examples for debriefing teamwork learning objectives** | | | |
| --- | --- | --- | --- |
| **Roles and Responsibilities** | | | |
| Debriefer Script | Reference Material | | Instructor Notes |
| · Let’s talk about how you functioned as a team.  · From my perspective it looked like you (*did/did not) have* a clear team leader and defined team roles. I think this is (*great/concerning)* because clear team roles can help a team function smoothly- improving how quickly interventions take place and reducing errors.  o How did you function as a team?  o What did you think about your roles? | Team leader  § Clear direction, coordination, timely interventions  § Foot of patient    Airway MD  § Manage airway  § Head of patient    Survey MD  § Primary, Secondary assessment, pulses, reassessments  Nursing roles  § Medication Prep (draw-up meds)  § Medication Admin (give meds)  § Documenting (time keeper) | |  |
| **Brief and Huddle** | | | |
| Debriefer Script | | Reference Material | Instructor Notes |
| I noticed that your team *(did/didn’t/took a while to)* (*brief* *prior to the initial patient assessment/huddle after the initial evaluation).* I thought this was (*great/could have helped you work better as a team*) in order to facilitate patient care.  · What *(helped/hindered)* your team from (*briefing/huddling*)?  · How did that impact your team?  · What could your team have done differently?  · How can you make sure that *(does/doesn’t*) happen again? | | The goal of a brief/huddle is to create a shared mental model. Assure all team members know what the working diagnosis is, management priorities and next steps in care.   - Everyone on the team is responsible for making this happen. Anyone can ask for a brief/huddle. Brief/huddle is usually led by team leader. - If one team member doesn’t know what’s up or what’s next- s/he is probably not alone. |  |
|  |  |  |  |

| **Directed call out** | | | |
| --- | --- | --- | --- |
| Debriefer Script | Reference Material | | Instructor Notes |
| I noticed that you (*did/didn’t/intermittently*) used (*peoples names/roles/eye contact*) when (*calling out orders/asking for assistance*). I thought this was (*great/could have been more directed*) in order to facilitate communication.  · What did you notice about orders/questions that were asked?  · How did this impact your team? | Directed call out. A communication skill to assure that important orders/questions are specifically directed to one individual (rather than called out into the air).  Example:  § “Jonathan-What’s the SaO2%?”  § “Kim- Give normal saline 500 mL”  § “Team leader- she stopped responding to pain” | |  |
| **Closed loop communication/Check back** | | | |
| Debriefer Script | | Reference Material | Instructor Notes |
| I noticed that you used closed-loop communication *(consistently/ a lot/rarely)*. Closed-loop communication can be critical for catching errors and assuring that *(information/an order/a request)* is heard.  · How were the communication loops in the team?  · How did that impact your team?  · Has anyone seen problems with this in a patient resuscitation?  · Has anyone seen closed loop communication prevent an error?  · How could you do it differently next time? | | Closed loop communication/check back is a strategy that requires verification of information. This enables the sender of the message to verify it has been heard and heard correctly. It enables the receiver to confirm what they heard is correct.  § Team leader “Call for x-ray”  § Float nurse “calling technician for an x-ray”  § Team leader “correct” |  |
|  |  |  |  |

**Bacterial Tracheitis Medical Management Evaluation/Debriefing Form**

This checklist identifies core medical management /technical skills. It’s hard to discuss more than 3 of these during one debriefing session. We recommend focusing on 2-3 of these issues.

**Initial Evaluation □** Done Well **□** Needs Work

Specific comments: _____________________________________________________________________

_____________________________________________________________________________________

*Discussion Points: What did you think of the initial evaluation of this patient? What could you do differently?*

**Recognition of bacterial tracheitis □** Done Well **□** Needs Work

Specific comments: _____________________________________________________________________

_____________________________________________________________________________________

*Discussion Points: What did you consider in your differential? What (went well/could have gone better) to help your team consider additional etiologies? How did your team (succeed/miss) identifying bacterial tracheitis?*

**Initial management of bacterial tracheitis □** Done Well  **□** Needs Work

Specific comments: _____________________________________________________________________

_____________________________________________________________________________________

*Discuss Points: What are the priorities for initial management of bacterial tracheitis? (ABCDE’s, racemic epinephrine, neck xray, targeted medications, anticipate risk for decompensating).What is the management of bacterial tracheitis? What are the risks/benefits related to PIV placement in young pediatric patients with stridor (potential worsening vs appropriate treatment)?*

**Reassessment of breathing after interventions □** Done Well **□** Needs Work

Specific comments: _____________________________________________________________________

_____________________________________________________________________________________

*Discuss Points: How did the patient’s breathing change after your interventions? What tests would be helpful? Consider neck x-ray, repeat racemic epinephrine, when refractory add intravenous antibiotics. How was the airway managed (OR vs ED)? Are there steps prior to intubation that could have been utilized (adjunct airways)?*

**Bacterial Tracheitis Teamwork and Communication Evaluation**

This checklist identifies core teamwork and communication skills. It’s hard to discuss more than 3 of these during one debriefing session. We recommend focusing on 2-4 of these issues.

**Leader/Roles Identified & Maintained □** Done Well **□** Needs Work

Specific comments: ____________________________________________________________

____________________________________________________________________________

____________________________________________________________________________

*Discussion Points: What helped/hindered having clear leadership and roles?*

**Directed Call out □** Done Well **□** Needs Work

Specific comments: ____________________________________________________________

____________________________________________________________________________

____________________________________________________________________________

*Discussion Points: How were orders given- “Into the air” or directed at specific individuals? How did that impact you? How could they be delivered more effectively?*

**Check back/Closed loop communication □** Done Well **□** Needs Work

Specific comments: ____________________________________________________________

____________________________________________________________________________

____________________________________________________________________________

*Discussion Points: Describe closed loop communication.*

**Shared Mental Model □** Done Well **□** Needs Work

Specific comments: ____________________________________________________________

____________________________________________________________________________

____________________________________________________________________________

*Discussion Points: How did team members share information/working diagnosis/management plan ((brief/huddle)?*
