# Supplementary material for: Pediatric Emergency Medicine Simulation Curriculum: Bacterial Tracheitis
Source: MedEdPORTAL. 2020 Aug 26;16:10946. doi: 10.15766/mep_2374-8265.10946 (PMC7449579; doi:10.15766/mep_2374-8265.10946)
Supplement: Supplementary file 1 — Bacterial Tracheitis Simulation Case.docxEnvironmental Preparation.docxCritical Action Checklist.docxSoft Tissue Neck X-Rays.docxChest X-ray.docxCommunication Glossary.docxDebriefing Guide.docxTeaching Handout.pdfEvaluation Form.docx [file mep_2374-8265.10946-s001.zip › H. Teaching Handout.pdf]

BACTERIAL TRACHEITIS

PEARLS:

1. Don't assume all stridor is croup
   a. Consider neck x-rays in an ill appearing child that does not improve with racemic epinephrine
2. If needing additional racemic epinephrine and / or looks sicker…think of tracheitis
3. High fever at the very start of illness and unwell appearing …. think of tracheitis
4. Otherwise healthy kids get tracheitis
   a. Particularly older healthy (and immunized) kids who seem to have croup
5. If you have thought of tracheitis twice and not sure, call otolaryngology

What is it? An infection of the tracheal soft tissues

Symptoms/Signs

- Fever (common but not always)
- Stridor
- Cough
- Respiratory distress
- May have drooling

Common bacterial pathogens:

- Staphylococcus aureus
- Streptococcus pneumococcus
- Group A streptococcus
- Alpha hemolytic streptococcus
- Moraxella catarrhalis
- Haemophilus influenzae

Predisposed to this by: viruses…such as Influenza (this patient ended up having influenza B)

- Influenza A (most common)
- Influenza B
- Respiratory Syncytial Virus
- Parainfluenza
- Measles
- Enterovirus

Where do you see this most commonly?

- Patients with artificial airways such as tracheostomies

But what about kids without tracheostomies?

- Usually have a prodrome of viral symptoms for a few days before developing stridor/dyspnea
- BUT, some pediatric patients have a rapid progression to acute respiratory distress within 24 hours of minor upper respiratory infection symptoms. These patients appear toxic**.**

Diagnosis

- Neck x-rays
  - Subglottic irregularities
- Bedside scope
  - Membranous exudate

Differential Diagnosis

1. Croup
2. Mediastinal Mass
3. Epiglottitis
4. Diphtheria
5. Anatomical abnormalities

Treatment

- Maintain the airway!
- Antibiotics-depends on the pathogen (if the patient is immunized)
  - Vancomycin or Clindamycin PLUS
  - Ceftriaxone or Cefotaxime or Ampicillin-sulbactam

Hopkins, A., et al., Changing epidemiology of life-threatening upper airway infections: the reemergence of bacterial tracheitis. *Pediatrics*, 2006. 118(4): p. 1418-21

Graf, J., Stein F. Tracheitis in pediatric patients. *Seminars in Pediatric Infectious Diseases*, 2006. 17(1):11-13.

Kuo CY, Parikh SR. In Brief: Bacterial Tracheitis. *Pediatrics In Review*, 2014. 35(11): 497-499.
