# Supplementary material for: Pediatric Emergency Medicine Simulation Curriculum: Bacterial Tracheitis
Source: MedEdPORTAL. 2020 Aug 26;16:10946. doi: 10.15766/mep_2374-8265.10946 (PMC7449579; doi:10.15766/mep_2374-8265.10946)
Supplement: Supplementary file 1 — Bacterial Tracheitis Simulation Case.docxEnvironmental Preparation.docxCritical Action Checklist.docxSoft Tissue Neck X-Rays.docxChest X-ray.docxCommunication Glossary.docxDebriefing Guide.docxTeaching Handout.pdfEvaluation Form.docx [file mep_2374-8265.10946-s001.zip › I. Evaluation Form.docx]

**Appendix I**: Bacterial Tracheitis Simulation Evaluation Form

**Instructor(s):** __________________ **Date:**  ____________

**Case Presented:** Pediatric Emergency Medicine Simulation Curriculum: Bacterial Tracheitis

**Circle one**: medical student/resident/fellow/attending (specialty:______), nurse, other:_______

|  | Strongly  Disagree | Disagree | Neutral | Agree | Strongly  Agree |
| --- | --- | --- | --- | --- | --- |
| 1. This case presented during the simulation is relevant to my work. | 1 | 2 | 3 | 4 | 5 |
| 1. The simulation case was realistic. | 1 | 2 | 3 | 4 | 5 |
| 1. This simulation case was effective in teaching basic resuscitation skills. | 1 | 2 | 3 | 4 | 5 |
| 1. I was able to practice assessing and emergently managing airway, breathing, and circulation. | 1 | 2 | 3 | 4 | 5 |
| 1. I can formulate a systematic approach (i.e. differential diagnosis) to the evaluation and management of pediatric stridor. | 1 | 2 | 3 | 4 | 5 |
| 1. I feel comfortable describing the signs and symptoms of bacterial tracheitis in a pediatric patient. | 1 | 2 | 3 | 4 | 5 |
| 1. The simulation allowed me to practice teamwork using principles of crisis resource management. | 1 | 2 | 3 | 4 | 5 |
| 1. I feel confident in constructing a disposition plan for a pediatric patient with bacterial tracheitis after stabilization in the emergency department. | 1 | 2 | 3 | 4 | 5 |
| 1. The debrief promoted reflection and team discussion. | 1 | 2 | 3 | 4 | 5 |
| 1. The facilitators created a safe environment for discussion and exploration. | 1 | 2 | 3 | 4 | 5 |

Can you list/describe 1 or more ways this session will change how you do your job?

How could we improve this simulation?

Additional Comments:
